# Notes on the Philippines, General and Medical

**Published:** 1902-06

**Authors:** L. B. Grandy

**Affiliations:** Major and Surgeon, U. S. Volunteers


### NOTES ON THE PHILIPPINES, GENERAL AND MEDICAL.

By L. B. GRANDY, M.D.,

Major and Surgeon, U. S. Volunteers.

For some time I have intended sending an article to THE JOURNAL-RECORD from these distant shores, but my good intentions have produced no results as yet, partly from lack of time, and partly, I suppose, from that lack of energy which more or less severely attacks every one in the tropics.

I shall attempt to present only some running notes on the Philippines, based upon personal observation and experience extending over nearly three years, rather than a detailed discussion of some particular subject. I believe the kind of paper proposed will be of more general interest than one more restricted in scope.

A few words, first, of general character about the islands and the people. Geographically, the Philippines comprise a chain of about eight hundred islands lying in a north and south direction and embracing about the same degrees of north latitude as Central America in the western hemisphere. Luzon and Mindanao are the two largest and together are about equal in area to all our New England States. Other important islands are Samar, Panay, Cebu, Leyte and Negros. Manila (on Luzon) is the largest city and capital city of the whole group, with a population of about 300,000. Iloilo (on Panay) is the next city.

The total population of the Philippines is approximately 8,000,000, representing three distinct races, the Malay, the Indonesian and the Negrito, which in turn include about seventy tribes. Of the latter the most important are the Visayans, the Tagalogs, the Bicols, the Ilocanos, the Pangisinans, the Pampangas and the Mohammedan Moros. Each tribe has its own language or dialect. The Negritos, or true blacks, are supposed to be the aborigines of the islands and are rapidly becoming extinct. The predominating tribes are the Visayans and Tagalogs (or Tagalos) numbering respectively 2,000,000 and 1,650,000.

By Filipino, in this paper, will be understood especially the

Tagalo type, because it is with this type that the writer is more familiar. These are the ones, aided and abetted by the Visayan, that have fomented insurrections and kept other tribes in terror. For ways that are dark he is a close second to the heathen Chinee. He is medium sized, copper colored, beardless, with black eyes and straight black hair. His lips are not so thick nor his nose as flat as our Southern negro's. His character is something unknown and unknowable. Priests who have had the most intimate relations with the native for years have admitted their total inability to understand his habits of thought and motives of action. He works only enough to supply his necessities, which are few, and takes no thought for the morrow. Rice, fresh cigarettes and betel-nut are his chief wants, with a little clothing. He is a born liar, and stealing is an agreeable and easy means of support. They rarely laugh or joke. They accept sickness or wounds with utter unconcern and await death with the indifference of a stoic. I have treated a number of wounded Filipinos and have found them almost invariably patient and uncomplaining. Some time ago one was brought into hospital with five gunshot wounds, and while I was amputating a finger, without an anesthetic, he coolly inquired if there was any objection to his smoking a cigarette. Their only sports are cock-fighting and gambling at cards; the former principally by men, the latter often by women. They are masters in the art of dissimulation and experts in concealing their thoughts. Your Filipino servant will profess the most loyal friendship and serve you well for years, and one day will rob you of anything lying around loose or carve you with his faithful bolo for a little money. Right in this town the Presidente (or Mayor) and the town Council several months ago took the oath of allegiance to the United States and assured us of their sincere devotion to America, but a few months later some papers were captured in an insurgent camp near here with items showing how much these very men were contributing to the insurgents. Gratitude for favors is the exception, and most of their tribe languages have no word for "thank you." A Filipino man will be ever so jealous of his wife's fidelity, but his daughter's character never causes him a thought. Both men and women are cleanly of their persons and dress, generally, but the pigs and chickens are all but members of the household and their premises are marvels of filth. The native half-breed is still worse. This odd combination of Filipino and Chinese (usually) is not a success. Those well acquainted with the species will tell you that he inherits the good qualities of neither parent but the vices of both.

Be it said however to the credit of the Filipino that he is sober, hospitable, slow to anger and kind to his family usually. I have seen only one intoxicated during my residence in the islands. It would be uncharitable not to make due allowances for the adverse circumstances in which the Filipino has spent his national life. His shortcomings are not all of his own making. They are the logical fruits of his environment, of the neglect of his paternal government for two hundred years, and of the ignorance which the Catholic church has forced upon him. The sins of omission and commission against the Filipino on the part of both the Spanish government and of the Roman church would, of themselves, fill a book. This is the cause of the various uprisings that have agitated the islands off and on for the last fifty years. The greed and avarice and arrogance and immoralities of the Spanish Friars, their supercilious and contemptuous treatment of the natives, finally became intolerable even to these long-suffering people; and as the civil government was itself controlled by the church and refused all petitions for reform, the only alternative was Revolution to the tune of "Down with the Friars." These priests systematically extorted money from the people in fees and robbed them of their lands under the pretext of collecting legitimate dues for the church, and thus became wealthy landholders of some of the most valuable properties in the islands. These are the "Friar Lands" which the Secretary of War has very wisely urged upon the attention of Congress, representing about 400,000 acres and valued at $6,000,000. They have disgusted the moral sense of the people by the practice of concubinage in spite of their religious vows, and the illegitimate fruits of their broken vows may be recognized anywhere.

The vast majority of Filipinos know absolutely nothing of liberty and independence, and probably care less. It is not to be expected that they should know. It has always been the policy of their rulers, civil and religious, to keep them from knowing. And it is entirely immaterial to them what government they are under, provided they are allowed to follow their customary pursuits unmolested and are not taxed to death. But to give self-government to such a people, expecting them to know what to do with it, would be about as sensible as presenting a telescope to a monkey expecting new light on the canals of Mars.

The best specimen of the Tagalo which has been produced, and one justly entitled to be called a patriot and a Moses to his race, was José Rizal, whose career may be appropriately referred to here, because he was distinguished as a physician as well as a leader of thought among his people. He is known as the Filipino martyr, a martyr to his convictions, and his memory is reverenced by all except that bigoted clerical clique that demanded his death. As a youth he obtained such education as the Jesuits' school in Manila could give him, and then, with a determined ambition, he decided to complete his studies in Europe. He graduated in medicine and philosophy at the University of Madrid and took postgraduate courses in Paris and Germany. While there he also studied closely European society and systems of government, and was led by comparison to deplore the woeful conditions existing in his own native islands. Then and there was born his patriotic ambition to "save his country from the baneful influence of the Spanish Friars who held the civil and military governments under their tutelage." He wrote a novel entitled *Noli me Tangere*, with a sequel, *El Filibusterismo*, which was an "exposé of the arrogance, the despotism and the immoralities of the friars in their relations with the natives." (I have presented copies of these books to the Carnegie Library, Atlanta.) He was the author also of some pamphlets of similar nature. After that the friars marked him for their victim. He returned to the Philippines, but was forced to leave. They hounded him from place to place and abused and persecuted his family. The civil governor authorized him to return to Manila, but the clericals had him arrested as soon as he arrived. He was tried and convicted on some trumped-up charges and deported to a little town on the island of Mindanao. It should be stated that the civil government, General Blanco commanding at that time, never considered Rizal guilty of any crime, and resisted the efforts of the clericals to persecute him, but without avail. After four years in Mindanao he volunteered his services to the Spanish government as surgeon for duty in Cuba. His offer was accepted, and in September, 1896, he sailed for Spain, intending to go on to Cuba. The priests doubtless believed that he was about to escape from their clutches, so upon his arrival in Spain he was again arrested by their command and ordered back to Manila for further humiliations. He was charged with *sedition and rebellion,* although he had been a prisoner under guard for years. An impartial writer who was in Manila at the time says that "no reliable testimony could be brought against him, . . . but he was condemned beforehand to ignominious death as a traitor." "What is death to me?" he said. "I have sown the seed, others are left to reap." The execution took place on the Luneta, Manila's fashionable driveway, December 30, 1896. "An immense crowd witnessed in silent awe this sacrifice to priestcraft. The friars were present *en masse,* many of them smoking big cigars, jubilant over the extinction of that bright intellectual light which, alas, can never be rekindled."—(*Foreman.*)

During the brief spells when Rizal was not being molested he was engaged in the practice of his profession and in literary pursuits. He was esteemed as a skillful physician and surgeon, and he performed successfully a number of important operations on the eye which contributed much toward his professional fame. He was ever loyal to Spain, his only crime apparently being a desire to "clip the wings" of the clergy, so to speak; to curtail their authority in secular affairs. To an autocratic, intolerant Catholicism, whose power none had dared to question, this was an unpardonable insult, to be avenged only by death. But if Rizal could return to Manila to-day he could rejoice in the fulfilment of his *ante-mortem* prediction. He would see the friars shorn of their power, their occupation narrowed down to religious duties in their churches and schools. He would see preparations well under way for a monument to himself which will be for a token that the rule of the clergy is over, and that they no longer hold the Philippines under their sacred thumbs.

As to climate, this is not so hot and disagreeable as most people imagine. The year is conveniently divided into the wet season (June to October, inclusive), the cool and dry season (November to February, inclusive), the hot and dry season (March to May, inclusive). By wet season is not meant a daily downpour of rain, though I have once seen this occur for ten days in succession. On the other hand there may be several continuous days of bright hot weather in the midst of the wet season. From June to October, however, it may rain any time, and a pleasant sunny day can become wet and sloppy on very short notice. A rain is very exceptional in other seasons. The temperature varies little from season to season, remaining close around 80 degrees in the shade on an average the year round. Daily records kept at my hospital show that during the hot weather in the States last summer when the thermometer was reading 95 to 100 every day, it did not go above 88 here. And we are 625 miles farther south than Havana. The tabulated records may be interesting:

MEAN DAILY TEMPERATURES AT LIPA, BATANGAS, P. I., FOR 1901.

| | 7 A.M. | 3 P.M. |
|---|---|---|
| January | 71.71 | 80.45 |
| February | 70.82 | 79.43 |
| March | 72.65 | 80.55 |
| April | 76.83 | 84.17 |
| May | 78.50 | 85.57 |
| June | 79.55 | 85.15 |
| July | 77.68 | 81.50 |
| August | 77.81 | 81.71 |
| September | 77.80 | 81.67 |
| October | 76.71 | 81.42 |
| November | 75.93 | 80.60 |
| December | 72.60 | 77.90 |

The direct rays of the sun about midday, however, are excessively hot, and prolonged outdoor labor at that time very trying, almost impossible for an American. Sunstroke, however, practically does not occur.

I do not see any good reason for considering the Philippines especially unhealthy, speaking, of course, only for the parts that I am familiar with, provided one observes common-sense precautions about diet, drinking-water and clothing, and tries to avoid influences conducing to malaria. But the mortality among natives will always be great until they can be taught something of public sanitation and aroused out of their stupid indifference to filth. Living, as many of them do, in crowded "shacks," with pigs and chickens underneath, and the family privy within a few feet of the well and kitchen, the only wonder is that filth diseases have not been more frequent and fatal than they have. Typhoid fever is not as prevalent as one would naturally expect from the universal disregard of public cleanliness. Malarial fever (*calentura*, as the natives call it), beri-beri and gastro-intestinal disorders constitute the large majority of the sickness, the last class annually slaying its thousands of children. Of course proper feeding of infants is something unheard of. A Filipino child is allowed to eat anything he can bite or swallow.

I wish to say something about Philippine fruits. These are exceedingly varied, but many of them not pleasing to the American palate. There are fifty-seven varieties of banana. I have seen only six of the varieties myself, and three of them are very delicious. I have never seen these kinds at home, and have not seen here the variety commonly sold in the United States. The mango grows to perfection. The fruit is yellow, kidney-shaped, about the size of a large pear, and has a delicate and delicious flavor and odor peculiarly its own. The papaya, from which papoid or vegetable pepsin is derived, grows wild; its medicinal value in dyspeptic conditions is well known empirically to the natives. The fruit, about the size of a small canteloup, and somewhat of that flavor, grows in clusters at the top of a limbless tree, the leaves of which make a fair substitute for soap. Among others there are the orange, several varieties, and very good; the guava; the watermelon, very inferior; the bread-fruit, chico, santol, and others which natives enjoy but we do not.

Several varieties of well-known medicinal plants are found in the islands, besides a great number of indigenous plants with native names which I cannot classify under official names known to us, and which are believed by the natives to possess remarkable curative properties for many ailments. Among medicinal plants are the St. Ignatius palm, producing the ignatia bean (called here *cat-*

*balogan nut*), the source of nux vomica and strychnine; menispernum, an infusion of which is used by natives for ulcers and skin eruptions; artemisia, used as a stomachic tonic; chenopodium, a decoction of which is used as a diuretic, and a poultice of the leaves for skin eruptions and furuncles; tamarind, a mild laxative; stramonium, the dried leaves smoked with tobacco for asthma, and a poultice of the fresh leaves in oil used for rheumatism; cassia fistula, a mild purgative; chrysarobin, or goa powder, used in skin eruptions, and particularly for "dhobie itch," about which more will be said further on. Then there is the betel-nut (piper betel), which is well-nigh indispensable to natives of the middle and lower classes. The nut is supposed to possess sustaining and stimulating properties similar to the kola nut, and is a stomachic tonic. It is chewed with a little powdered oyster shell to neutralize the acidity, and stains the teeth deep red. Nearly all natives use it, of the classes mentioned, and when their teeth are lost and they can no longer chew, they grind it into a paste in a mortar, and by rolling the sweet morsel around over their toothless gums they are able to get the physiological effect and comfort just the same.

The favorite beverage of the native is *Tuba*, the sap of a species of cocoanut tree. This is extracted at certain seasons by incising the bark and placing vessels to collect it. The yield from one tree continues about two months. It is a clear, sweetish fluid, and is usually drunk fresh. If allowed to stand and ferment it becomes intoxicating. Cocoa-wine is made from the fermented juice by distillation. Usually a certain number of trees are reserved in cocoanut palm plantations for supplying tuba. These trees produce no nuts.

Only two varieties of poisonous snakes are found, with native names, for which I know of no English equivalent. They inhabit the rice fields and swamps, and their bite is said to be rapidly fatal. Boa-constrictors are sometimes met with, but are generally harmless. They are often kept about houses to destroy rats. The house newt, a small lizard, exists everywhere, running about the floors or walls or about a table at night where a light is burning, in search of small insects, such as moths or gnats. They are perfectly harmless. The greatest pest is a white ant, native name

*Anay*, and the amount of damage which it can produce in soft woods is something remarkable. All furniture has to be made of certain hard woods, such as Molave, a kind of mahogany, of which fortunately there are many varieties in abundance. I have seen walls or partitions in houses so honeycombed by this ant that I could thrust my finger through them as easily as through paper. The ordinary white pine goods-box is pie to them. They are also fond of writing-paper, books, straw hats, shoe-uppers, etc. They even consume the supports of buildings, rendering them unsafe, and sometimes houses have to be torn down and rebuilt.

I now propose to take up briefly some of the diseases, more or less incident to the country and climate, and finally it will be my purpose to discuss more at length the soldier's worst enemies in the tropics, malaria and dysentery.

*Smallpox.*—This disease has played havoc among the natives in times past, the last severe epidemic having occurred about ten years ago. The Spanish government at that time instituted compulsory vaccination, but as the native had to pay one dollar to be vaccinated, all doubtless avoided it who could. One can see here a surprising number of pock-marked people, probably one-fourth of the population over ten years of age. The natives in their ignorance do not dread the disease; those who have it merely take to the house for a few days, or perhaps not even that. Cases have been recognized by army surgeons walking about the streets, or working in their shops or at their booths in the market. It creates great consternation among them when we hurry these people off into an isolated "shack" and put a guard over them. The last case of smallpox in this town occurred in an American soldier about one year ago. All over the islands sporadic cases and some deaths have occurred among the soldiers, but so far as I am aware they have nowhere run into a real epidemic. This is due, I believe, to the thorough system of vaccination prevailing in the service. Both officers and men out here have been vaccinated over and over again. If there is anything in vaccination, I do not believe it would be possible for smallpox to do much damage among our troops. One of the first things done after the capture of Manila was to establish near there a vaccine farm, and others have since been established in other places. The animals used are young caribous, or water buffaloes, peculiar to these islands. Compulsory vaccination of the natives has been going on for some time, without the dollar annex, however, which the Spanish enjoyed, and I presume nearly the whole islands have been well canvassed. We are just finishing up the work here in Lipa, population about 30,000. Four natives are employed and instructed how to do the work, under a guard of two soldiers, and they have orders to vaccinate everybody except those with pock-marks. Up to to-day they have vaccinated about 20,000. Each vaccinator is paid $15.00 per month. As long as it is free the natives do not object. Native physicians tell me that the disease often runs a very mild course here, and I judge from the few cases that I have seen among Filipinos and the slight marking, that this is true.

*Beri-Beri.*—This is very common among the natives; so much so that they all have some general knowledge of it and can often make a diagnosis themselves, just as the laity at home may recognize measles. Though beri-beri always exists there was a sudden severe outbreak about two years ago among some prisoners at Cavite, across the bay from Manila. At that place there is a beri-beri hospital which usually contains three or four hundred patients. It is the rarest thing for an American or European resident to have it. I have seen one soldier who had nearly every indication of the atrophic variety, but as he was under my observation only a few days I do not know if the diagnosis was confirmed by his subsequent history. The disease is seen in both the paraplegic and the edematous forms. In the first, one notices, in addition to the ataxic gait, anesthesia of the skin of the anterior tibial regions and of the dorsum of the feet, flabby and atrophic posterior tibial muscles and pain upon deep pressure over these muscles. The patella reflex is abolished. In the edematous cases the legs and feet become distended with a dropsical effusion as in acute Bright's disease; and the dropsy soon involves the arms, hands and face. Mixed cases also occur, presenting features of both varieties. In nearly all cases the heart is involved sooner or later. The patient dies from the cardiac complications, or from general exhaustion. Some of these patients make as distressing a picture as I have ever witnessed. Often bedridden for months, with bed-sores, and tissues water-logged or emaciated to skin and bone, perhaps unable to attend to their slightest personal wants, they doubtless welcome death as the end of a condition which they know is without a shadow of hope.

(To be concluded.)

*Lipa, P. I., March 24, 1902.*

